# Studienabschnitt „Vom Symptom zur Erkrankung“ – Schritte in ein integriertes Zahnmedizincurriculum

**DOI:** 10.1007/s00103-023-03800-6

**Published:** 2023-11-10

**Authors:** Rüdiger Lemke, Christine Mirzakhanian, Susanne Sehner, Natascha Bruhn

**Affiliations:** 1 Zentrum für Zahn‑, Mund- und Kieferheilkunde, Poliklinik für Parodontologie, Präventive Zahnmedizin und Zahnerhaltung, Universitätsklinikum Hamburg-Eppendorf, Martinistr. 52, 20246 Hamburg, Deutschland; 2 Poliklinik für Zahnärztliche Prothetik, Universitätsklinikum Hamburg-Eppendorf, Hamburg, Deutschland; 3 Institut für Medizinische Biometrie und Epidemiologie, Universitätsklinikum Hamburg-Eppendorf, Hamburg, Deutschland; 4 Poliklinik für Kieferorthopädie, Universitätsklinikum Hamburg-Eppendorf, Hamburg, Deutschland

**Keywords:** Zahnmedizin, Integration, Wissenschaftsstrang, Kommunikation, Früher Patient:innenkontakt, Dentistry, Integration, Science track, Communication, Early patient contact

## Abstract

In Anlehnung an den am Universitätsklinikum Hamburg-Eppendorf bereits etablierten Modellstudiengang Humanmedizin (iMED) wurde das zahnmedizinische Ausbildungskonzept grundlegend überarbeitet und der Modellstudiengang Zahnmedizin (iMED DENT) entwickelt. Hierbei wurden Reformziele wie Interdisziplinarität zwischen Zahnmedizin und Medizin, früher Patient:innenkontakt, psychosoziale und kommunikative Kompetenzen sowie eine wissenschaftliche Orientierung festgelegt. Der zweite Studienabschnitt „Vom Symptom zur Erkrankung“ im 2. und 3. Studienjahr stellt das Bindeglied zwischen der „Normalfunktion“ im 1. Studienjahr und dem letzten klinischen Ausbildungsabschnitt „Therapie“ dar. Der modulare Aufbau des Modellstudiengangs ermöglicht es, Themen repetitiv aufzugreifen und zu vertiefen sowie in der präklinisch praktischen Ausbildung zahnärztliche Fertigkeiten als Vorbereitung auf die Behandlung von Patient:innen zu verfestigen. Die zahnärztliche Prophylaxe, das Assistieren in der Klinik und die Befunderhebung bei Patient:innen schaffen in diesem Ausbildungsabschnitt den frühen Bezug zur Praxis. Neu integriert sind der Wissenschafts- und Kommunikationsstrang, die ebenfalls modular und in enger Verzahnung mit der Zahnmedizin stattfinden. Die bisherigen Ergebnisse der jährlichen Lehrevaluationen der ersten 3 Kohorten des Modellstudiengangs sprechen für eine erfolgreiche Umsetzung, zeigen aber auch, dass die kontinuierliche Weiterentwicklung und Verbesserung des Konzeptes anzustreben sind.

## Abstract (en)

Based on the integrated medicine program (iMED) at the University Medical Center Hamburg-Eppendorf, the dental education concept was fundamentally revised and an integrated dentistry program (iMED DENT) was developed. During this process, reform goals such as interdisciplinarity between dentistry and medicine, early patient contact, and psychosocial and communicative competencies as well as a scientific orientation were defined. The second study section “From Symptom to Disease” during the second and third years of study form a link between “Normal Function” in the first year and the final training section “Therapy”. The modular structure of the integrated dentistry program allows topics to be taken up repetitively and deepened as well as strengthen dental skills during the preclinical practical training in preparation for patient treatment. Here, the implementation of dental prophylaxis, assisting in the clinic, and the assessment of patients’ findings create an early link to the practice. Newly integrated are the science and communication tracks, which are also modular and closely interlinked with dentistry. So far, the results of the first three cohorts indicate a successful implementation of the new concept, but it should be continuously developed and improved.

## Einleitung

„Vom Symptom zur Erkrankung“ betitelt den mittleren von 3 Studienabschnitten des Modellstudiengangs Zahnmedizin (iMED DENT) und bildet eine Brücke zwischen den Abschnitten „Normalfunktion“ (siehe auch Beitrag von Bender et al. in diesem Themenheft) und „Therapie“. Dieser Studienabschnitt besteht aus 8 Modulen, die in 4 aufeinanderfolgenden Semestern unterrichtet werden (siehe auch Beitrag von Guse et al. in diesem Themenheft). Die ersten Module bestehen aus rein präklinischen Anteilen und werden bis zur Erlangung der Behandlungskompetenz zunehmend durch klinische Ausbildungseinheiten erweitert.^1^

In den Lehrevaluationen des Regelstudiengangs (RSG) wurden fehlende Zusammenhänge der allgemeinmedizinischen Fächer zur Zahnmedizin kritisiert (Freitextkommentar 8. Semester RSG Sommersemester 2022: „Jedes Fach macht so das ‚eigene Ding‘ und ist gar nicht abgestimmt mit den anderen Fächern.“)^2^. Im jetzigen iMED DENT werden daher die Ätiologie, Pathogenese, Diagnostik und Therapie zahnmedizinischer und für die Zahnmedizin relevanter medizinischer Erkrankungen fachinterdisziplinär gelehrt. Hierzu wurden die Schnittstellen zwischen den Fachbereichen identifiziert und die Lehrinhalte der Unterrichtsfächer unter Berücksichtigung des Nationalen Kompetenzbasierten Lernzielkatalogs Zahnmedizin (NKLZ) vernetzt.

Ziel dieses 2‑jährigen Studienabschnittes ist es, die Studierenden in ihren theoretischen, praktischen und kommunikativen Fähigkeiten auf die synoptische Patient:innenbehandlung im dritten Studienabschnitt vorzubereiten. Der frühe Patient:innenkontakt im Modellstudiengang soll den Studierenden ermöglichen, die Lehrinhalte mit stärkerem klinischen Bezug zu erlernen. Dazu gehören gegenseitige Befundaufnahmen, Abformübungen und professionelle Zahnreinigungen sowie die Herstellung und Eingliederung einer Aufbissschiene. Am Ende des Studienabschnitts steht die Befundaufnahme bei Patient:innen, die im letzten Studienabschnitt „Therapie“ von den jeweiligen Studierenden weiterbehandelt werden. Parallel zu diesen ersten klinischen Übungen wird der longitudinale Ausbildungsstrang psychosozialer und kommunikativer Kompetenzen durchlaufen, in dem die Studierenden in der Kommunikation mit Patient:innen geschult werden.

Ein weiterer Schwerpunkt ist die Vermittlung wissenschaftlicher Grundkenntnisse. Die Studierenden erlangen die Befähigung, wissenschaftliche Literatur zu verstehen und zu interpretieren, um die Grundlagen der evidenzbasierten Medizin kennenzulernen.

Am Ende des Studienabschnitts wird die klinische Behandlungsreife, d. h. die Befähigung zur Patient:innenbehandlung, durch die bestandene „Z2 Prüfung“ erteilt.

Im Folgenden berichten wir über die Modulblöcke (C, D, E, F, G und S) und Prüfungen, die im Studienabschnitt „Vom Symptom zur Erkrankung“ (2. und 3. Studienjahr) stattfinden.

## Modulstruktur und Modulinhalte

Die Modulblöcke C, D, E, F, G und S haben jeweils ein übergeordnetes Thema und bestehen aus maximal 3 Modulen, die im mittleren Studienabschnitt beginnen und im Sinne der Lernspirale im finalen Studienabschnitt „Therapie“ fortgeführt werden. Ein Semester besteht aus jeweils 2 Modulen à 7 Wochen (siehe auch Beitrag von Guse et al. in diesem Themenheft).

### Modulblock C

Der Studienabschnitt beginnt mit dem Modulblock C „Infektionen, Entzündungen und Prävention“. Im Vordergrund stehen zahnmedizinische Erkrankungen, deren Prävention und Therapie sowie das Grundverständnis der Bakteriologie. Zusätzlich werden erste Grundlagen von zahnmedizinisch relevanten Allgemeinerkrankungen vermittelt. Die praktischen Anteile, wie gegenseitige Mundhygieneeinweisungen und professionelle Zahnreinigungen, sollen das Verständnis der Studierenden für pathogenetische Zusammenhänge verschiedener Erkrankungen fördern (NKLZ Kapitel Z12, Z19 und Z23).

#### Modul C1

Schwerpunkte des Moduls sind die Zusammensetzung und Struktur der Bakteriologie der Mundhöhle vom physiologischen zum pathologischen Zustand. Als Ursache zahnmedizinischer Erkrankungen und anderer Infektionen steht sie fächerübergreifend im Mittelpunkt des Moduls. Aus der Zahnmedizin werden die klinischen Aspekte von Erkrankungen wie Karies und Gingivitis sowie deren Prävention gelehrt. Die Fächer Biochemie und Mikrobiologie geben interdisziplinär ein Überblick über die Diagnostik und den Stoffwechsel der Bakterien, die in Zusammenhang mit allgemeinmedizinischen und zahnmedizinischen Erkrankungen stehen. Ergänzend werden die pharmakologischen Grundlagen zu Antibiotika und deren Pharmakodynamik vermittelt.

Ein erster Einblick erfolgt in klinische Aspekte zahnmedizinisch bedeutsamer Erkrankungen aus den Bereichen Herz/Kreislauf, Atmung und Stoffwechsel. Auch hier wurde in der Entwicklung des iMED DENT darauf geachtet, dass der Schwerpunkt auf den zahnmedizinisch bedeutsamen und eher häufigeren allgemeinmedizinischen Erkrankungen liegt. Dies ist in diesem Modul zum ersten Mal relevant, da die Studierenden in Theorie und Praxis die zahnärztliche Prophylaxe erlernen, vor deren Durchführung sie eine gegenseitige Anamnese aufnehmen. Sie erstellen Präventionskonzepte, instruieren sich gegenseitig in der Mundhygiene und führen untereinander professionelle Zahnreinigungen durch.

In der praktischen präklinischen Ausbildung an Phantommodellen erlernen die Studierenden in diesem Modul die Therapie von Karies. Im theoretischen Teil werden begleitend sowohl die Eigenschaften als auch die Verarbeitung von Füllungsmaterialien (Komposit, Keramik, Gold) sowie deren spezifische Präparationsformen vermittelt.

Die Lernspirale im Bereich psychosozialer und kommunikativer Kompetenz im Umgang mit Patient:innen beginnt in diesem Modul mit Themen wie Emotion und Motivation, Lernen, subjektive Krankheitstheorien sowie Gesundheits- und Krankheitsverhalten. Ein Teil der kommunikativen Ausbildung erfolgt über Simulationspatient:innen, da diese praxisorientierte Schulung einen hohen Lerneffekt bietet [1–3]. Die Erarbeitung der Schulungsthemen und die Schulung der Simulationspatient:innen wird interdisziplinär durch die zahnmedizinischen Fächer und die Institute für Medizinische Psychologie, Ethik und Allgemeinmedizin durchgeführt.

#### Modul C2

Im 4. Semester beginnt der zweite Teil der Lernspirale „Infektionen, Entzündungen und Prävention“. Die Ausbildungsinhalte im Modul C2 greifen die Themen aus dem Modul C1 auf und erweitern das zahnmedizinische Wissen der Studierenden um parodontale sowie endodontische Zahnerkrankungen und deren Therapie.

Die Ausbildung in der endodontischen und postendodontischen Therapie ist ein Beispiel für die synoptische Ausbildung der Studierenden: Während im RSG die Endodontie (gelehrt durch die Zahnerhaltung) und die postendodontische Versorgung (gelehrt durch die Prothetik) in unterschiedlichen Semestern liegen, unterrichten im Modellstudiengang beide Polikliniken im Sinne des synoptischen Behandlungskonzeptes diese zusammengehörigen Themen zeitlich und inhaltlich aufeinander abgestimmt.

Ein weiteres Beispiel der integrativen Ausbildung ist der Unterricht zur juvenilen idiopathischen Arthritis (JIA). In aufeinander aufbauenden Vorlesungen werden die klinischen Parameter der Erkrankung durch das Fach Kieferorthopädie und die molekularen Zusammenhänge durch das Fach Biochemie dargelegt. Ergänzend dazu wird die JIA durch das Fach Kinder- und Jugendmedizin in den allgemeinmedizinischen Kontext gesetzt.

### Modulblock D

Im Modulblock D stehen „Angeborene und erworbene Zahn- und Kieferdefekte und initialer Zahnverlust“ im Mittelpunkt. Die in den präklinischen Modulen D1 und D2 im Studienabschnitt „Vom Symptom zur Erkrankung“ erlernten zahnmedizinischen Inhalte werden in Modul D3 im klinischen Umfeld praktisch an Patient:innen angewandt (NKLZ Kapitel Z23a und Z23d).

#### Modul D1

In Modul D1 werden die erworbenen Basiskenntnisse zu übertragbaren Infektionen sowie die Präventions- und Hygienemaßnahmen bei der zahnärztlichen Behandlung aus dem Modul C1 durch Veranstaltungen der Mikrobiologie, Virologie und Hygiene fortgeführt.

Diese werden durch die zahnklinischen Fächer mit der synoptischen Befundaufnahme und der interdisziplinären Behandlungsplanung verknüpft und im Rahmen des frühen Patient:innenkontaktes an Kommiliton:innen praktisch unterrichtet und angewendet.

In weiteren Vorlesungen werden die Kenntnisse zu angeborenen und erworbenen Zahndefekten und deren Therapie in Form von Kronen zur Versorgung einzelner geschädigter Zähne vermittelt. Im praktischen Unterricht werden an Phantommodellen Zahnpräparationen, die provisorische Versorgung sowie digitale Abformungen durchgeführt und anhand einer computerbasierten Musterpräparation überprüft.

Anknüpfend an die klinischen Themen werden im Wissenschaftsstrang die Grundlagen der evidenzbasierten Medizin vermittelt (s. Abschn. 2.6).

#### Modul D2

In diesem Modul wird die Therapie der Einzelzahnlücke thematisiert und im präklinischen Kurs am Phantommodell durchgeführt. Hierzu werden von den Studierenden Zähne präpariert und eine provisorische Brücke konventionell hergestellt. Eine weitere Therapieoption bei Einzelzahnlücken ist ein dentales Implantat mit einer Krone. Hierzu werden in der Vorlesung die Grundlagen vermittelt und im präklinischen Kurs die analoge offene und geschlossene Implantatabformung geübt.

Zusätzlich wird eine Einzelzahnpräparation digital abgeformt und der CAD/CAM(„computer-aided design“/„computer-aided manufacturing“)-Arbeitsprozess durchlaufen. Die konstruierten Restaurationen werden in einem externen Labor aus 2 in der zahnärztlichen Praxis gängigen Werkstoffen (Oxidkeramik und edelmetallfreie Legierung) gefräst und anschließend am Phantommodell angepasst und eingesetzt. Hierzu werden die Oberflächen der Restaurationen nachbearbeitet, sodass sie den klinischen Anforderungen an Patient:innen entsprächen.

Die Fächer Mund‑, Kiefer- und Gesichtschirurgie, Zahnerhaltung, Rechtsmedizin und Kieferorthopädie lehren in aufeinander aufbauenden Veranstaltungen die Themen der Zahntraumatologie und dentoalveolären Chirurgie. Hierbei werden die Ursachen von Zahntraumata, die Beurteilung der Erhaltungswürdigkeit von traumatisierten Zähnen, deren fachspezifische und interdisziplinäre Therapie sowie rechtliche Aspekte vermittelt.

Passend dazu führt die zahnärztliche Radiologie den „Kurs und Praktikum der Zahnärztlichen Radiologie unter besonderer Berücksichtigung des Strahlenschutzes“ fort, der für den Erwerb der Fachkunde im Strahlenschutz erforderlich ist und in Modul F1 begonnen hat.

Im Rahmen der Lernspirale des Kommunikationsstranges wird die Gesprächsführung mit Schmerzpatient:innen und Patient:innen mit Zahnbehandlungsangst durch praktische Seminare mit Simulationspatient:innen unterrichtet. Diese Lehre wird interdisziplinär durch die zahnmedizinischen Fächer sowie die medizinische Psychologie, Allgemeinmedizin und psychosomatische Medizin entwickelt und ausgerichtet.

### Modulblock E

Im Zentrum des Modulblocks E „Zahn- und Kieferfehlstellungen“ stehen Befunderhebung, Diagnostik und Therapie wichtiger kieferorthopädischer Anomalien und interdisziplinärer Fälle. Zusätzlich wird der Fokus im klinischen Studienabschnitt „Therapie“ in den Modulen E2 und E3 auf die Behandlung von Patient:innen im Rahmen des synoptischen Behandlungskurses und das Chairside-Teaching gelegt (NKLZ Kapitel Z23h).

#### Modul E1

Konzeptionell stehen Befunderhebung und Diagnostik von Zahn- und Kieferfehlbildungen sowie die vielschichtige Verknüpfung medizinischer Grundlagenfächer mit zahnmedizinischen Lehrinhalten im Mittelpunkt des Moduls E1.

In seiner ursprünglichen und derzeit noch gültigen Form erlernen die Studierenden im Modul die Grundlagen der Kopfanatomie und des zentralen Nervensystems sowie biochemische Zusammenhänge des stomatognathen Systems. In den Vorlesungen und Praktika der Anatomie stellen die Studierenden ein Kopfpräparat her, um z. B. die Orbita, die Mundhöhle sowie wichtige Kopfarterien kennenzulernen. Außerdem wird anhand von Gehirn- und Rückenmarkspräparaten die Anatomie des zentralen Nervensystems vermittelt. Begleitend werden in Vorlesungen, Seminaren und Praktika der Neurophysiologie die Schmerzphysiologie, das auditorische und visuelle System sowie die periphere und zentrale Motorik gelehrt.

Abgerundet werden die medizinischen Grundlagen des Kopfsystems durch Veranstaltungen der Biochemie zu den Themen Stoffwechsel und Signaltransduktion, in denen u. a. die biochemischen Zusammenhänge von häufigen Zahnhartsubstanzanomalien wie der Molaren-Inzisiven-Hypomineralisation erläutert werden. Darauf aufbauend und im engen Bezug zur Biochemie stehen die fachübergreifenden Vorlesungen der zahnmedizinischen Fächer und der Humangenetik zu den verschiedenen Formen der Zahn- und Dentitionsanomalien und deren Therapiemöglichkeiten. Ergänzend hält die Mund‑, Kiefer- und Gesichtschirurgie gemeinsam mit den zahnmedizinischen Fächern Vorlesungen zum Thema „Interdisziplinäre Behandlungsplanung“.

Ein großer Teil des Moduls E1 wird von der Poliklinik für Kieferorthopädie ausgerichtet. In den Veranstaltungen wird die kieferorthopädische Röntgendiagnostik vermittelt und insbesondere die Analyse des Fernröntgenseitenbildes (FRS) erklärt und eigenständig durchgeführt. Zusätzlich vertiefen die Studierenden in den Seminaren die Befunderhebung und Diagnostik von Zahn- und Kieferfehlstellungen und erstellen in Vorbereitung auf die Inhalte der Z2-Prüfung Referate zu ausgewählten Themen.

Der praktische Teil des Moduls besteht aus einem kieferorthopädischen Kurs, in dem ein herausnehmbares kieferorthopädisches Gerät angefertigt wird.

Komplettiert wird das Modul durch Veranstaltungen des Kommunikationsstranges, in denen die medizinische Dokumentation vermittelt wird. Die kommunikativen Lernziele des Moduls sind das Lesen und Verstehen von Arztbriefen und wie deren Inhalte laienverständlich erklärt werden (NKLZ Kap. 5).

Die Studierenden der ersten und zweiten Kohorte, die das Modul E1 im Sommersemester 2021 bzw. 2022 besuchten, bewerteten das Modul negativ (1,9 bzw. 2,0 von 6,0 Punkten im Item „Mit dem Modul bin ich insgesamt zufrieden“; Tab. 1).

**Table 1:** 

Modul/Semester		„Mit dem Modul bin ich insgesamt zufrieden“		
Modul	Semester	*n* (Semesterstärke; Rücklauf in %)	Median	IQR
C1	WiSe 20/21	56 (64; 87,5)	4,5	4–5
C1	WiSe 21/22	56 (60; 93,3)	5	5–6
C1	WiSe 22/23	48 (58; 82,8)	5	4,25–5
D1	WiSe 20/21	55 (64; 85,9)	3	2–4
D1	WiSe 21/22	52 (53; 98,1)	3	2–4
D1	WiSe 22/23	50 (52; 96,2)	4	2,75–5
C2	SoSe 2021	60 (62; 98,4)	5	4–5
C2	SoSe 2022	50 (54; 92,6)	5	5–6
E1	SoSe 2021	40 (62; 64,5)	2	1–2
E1	SoSe 2022	51 (51; 100)	2	1–3
F1	WiSe 21/22	55 (59; 93,2)	5	4–5
F1	WiSe 22/23	47 (50; 94,0)	5	4–6
S	WiSe 21/22	54 (56; 96,4)	4,5	3,75–6
S	WiSe 22/23	49 (54; 90,7)	5	4–6
D2	SoSe 2022	44 (50; 88,0)	4	4–5
G1	SoSe 2022	46 (47; 97,9)	5	5–6

*n* Anzahl der rückläufigen Fragebögen, *IQR* Interquartilsabstand, *WiSe* Wintersemester, *SoSe* Sommersemester

**Figure 1:**
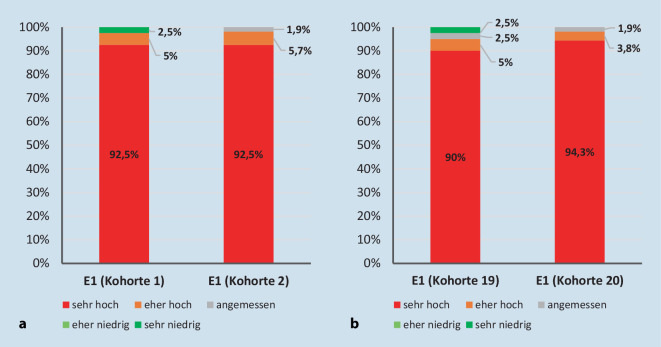

Die geringe Gesamtzufriedenheit lässt sich durch die als sehr hoch empfundenen inhaltlichen Anforderungen der 11 beteiligten Fächer und die damit verbundene zeitliche Auslastung erklären (Abb. 1).

Die einzelnen Lehrveranstaltungen hingegen wurden trotz hybrider Durchführung aufgrund der COVID-19-Pandemie mit wenigen Ausnahmen zufriedenstellend (> 3 Punkte) bewertet.

Aufgrund der hohen Prüfungslast, der hohen Anzahl beteiligter Fächer und der Evaluationsergebnisse nahmen die verantwortlichen Lehrenden eine umfangreiche Umstrukturierung der Lehrinhalte vor, indem diese zeitlich angepasst oder in andere inhaltlich passende Module (Modul D1 und C2) verschoben wurden. Eine fortwährende Anpassung des Moduls und kontinuierliche Optimierung der Stundenpläne werden weiterhin im Fokus der verantwortlichen Lehrenden stehen.

### Modulblock F

Das Modul F1 „Fortgeschrittener Zahnverlust und Zahnlosigkeit“ findet zu Beginn des 5. Fachsemesters im Studienabschnitt „Vom Symptom zur Erkrankung“ und das Modul F2 im 8. Fachsemester im Abschnitt „Therapie“ statt (NKLZ Kapitel Z22 und Z23d).

#### Modul F1

Im Modul F1 wird der frühe Patient:innenkontakt, der in vorangegangenen Modulen durch Übungen der Studierenden untereinander begonnen hat, durch das Assistieren bei höheren Semestern im Behandlungskurs des klinischen Studienabschnitts fortgeführt. Dies markiert einen Meilenstein der Lernspirale, in dem als Vorbereitung auf die Behandlung von Patient:innen die klinische Behandlungsumgebung kennengelernt wird und eine Einführung in die zahnärztliche Tätigkeit stattfindet.

Die Mund‑, Kiefer- und Gesichtschirurgie gibt – passend zum Modulthema – eine aus Vorlesungen und praktischen Übungen bestehende Einführung in die Zahnextraktion. Begleitend werden Schnitt- und Nahttechniken durch die Allgemeinchirurgie gelehrt.

Darauf aufbauend vermittelt die zahnärztliche Prothetik die Rehabilitation von zahnlosen und teilbezahnten Kiefern mit abnehmbaren Prothesen und die hierbei verwendeten Werkstoffe. Von der Indikation bis zur Nachsorge werden dabei alle Behandlungsschritte im praktischen Kurs und in der Theorie anhand praxisnaher Fallbeispiele erläutert.

Die zahnärztliche Radiologie führt die erste Unterrichtseinheit „Kurs und Praktikum der Zahnärztlichen Radiologie unter besonderer Berücksichtigung des Strahlenschutzes“ durch, welche in Modul D2 fortgeführt wird.

Durch die medizinische Psychologie wird mit Simulationspatient:innen die „Partizipative Entscheidungsfindung“ als Vorbereitung zu Therapieentscheidungen unterrichtet. Die Grundlagen des wissenschaftlichen Arbeitens aus vorhergehenden Modulen werden durch Vorlesungen und Seminare des Wissenschaftsstranges in Modul F1 vertieft (s. Abschn. 2.6).

### Modulblock G

Am Ende des Studienabschnittes „Vom Symptom zur Erkrankung“ und als Übergang in den klinischen Studienabschnitt beginnt der Modulblock G. Entsprechend dem Titel „Synoptische Behandlungsplanungen“ ist Modul G1 auf die Behandlung von Patient:innen ausgerichtet und sehr praxisorientiert. Im Modul G2, das im klinischen Studienabschnitt „Therapie“ liegt, werden komplexere Fälle in ihrer Gesamtheit erörtert, in Seminaren geplant und diskutiert (NKLZ Kapitel Z12b, Z19 und Z21).

#### Modul G1

Das Modul „Synoptische Behandlungsplanung I: Einfache Fälle“ wird in der zweiten Hälfte des 6. Semesters durchgeführt und wurde bisher nur von der ersten Kohorte durchlaufen. Von allen präklinischen Modulen ist G1 bisher am besten evaluiert worden (Tab. 1).

Zu den Lehrinhalten gehören präklinische praktische Übungen an den Simulationseinheiten und klinische Ausbildungselemente wie die Befunderhebung bei Patient:innen, die in den darauffolgenden klinischen Kursen behandelt werden. In gemeinsamen Seminaren durch die zahnärztliche Prothetik und Zahnerhaltung wird anhand von Patient:innenfällen die synoptische Behandlungsplanung mit den Abläufen der klinischen Behandlungsphasen erarbeitet. Ziel ist es, den Studierenden die Kenntnisse und Fertigkeiten zur eigenständigen Durchführung dieser Planungen auch bei eigenen Patient:innen im darauffolgenden ersten klinischen Semester zu vermitteln.

Zu den weiteren Übungen am Behandlungsstuhl zählen das Legen von Retraktionsfäden sowie Infiltrations- und Leitungsanästhesien der Studierenden untereinander. Die Theorie und Praxis dazu werden interdisziplinär durch die zahnmedizinischen Fächer und die Pharmakologie gelehrt. Die präklinischen praktischen Übungen an den Simulationseinheiten sind – im Sinne der Lernspirale – Wiederholungen erlernter Arbeitsabläufe, wie z. B. den digitalen Workflow von der Präparation bis zur frästechnischen Herstellung der Restaurationen zu vertiefen. Ergänzt wird das Modul durch den kieferchirurgischen Ausbildungsblock, in dem die Basisaspekte der Zahnextraktion und Nahttechnik vertieft werden.

Das Lehrkonzept in Modul G1 dient dazu, Behandlungsabläufe vor dem klinischen Ausbildungsabschnitt zu verfestigen, neue Parameter zu implizieren und gleichzeitig die Studierenden auf die im Anschluss stattfindende Z2-Prüfung vorzubereiten.

### Longitudinaler Wissenschaftsstrang und Modul S

Der longitudinale Wissenschaftsstrang soll den Studierenden die Möglichkeit bieten, wissenschaftliches Arbeiten in einem studienbegleitenden Curriculum zu erlernen und anzuwenden. Ziel ist es, den Studierenden zu vermitteln, sich als lebenslang Lernende zu verstehen und ihr professionelles Handeln durch stetiges Weiterlernen zu verbessern. Sie sollen befähigt werden, Verantwortung für ihre kontinuierliche Fortbildung zur Aufrechterhaltung und Weiterentwicklung zahnärztlicher Kompetenz zu übernehmen, indem sie ihren Entwicklungsstand in den einzelnen Kompetenzbereichen adäquat einschätzen, bewerten und gegebenenfalls passende Maßnahmen (wie berufsständige Fortbildungen oder eigene Literaturrecherche) wahrnehmen (NKLZ Kapitel Z6 und Z17).

**Figure 2:**
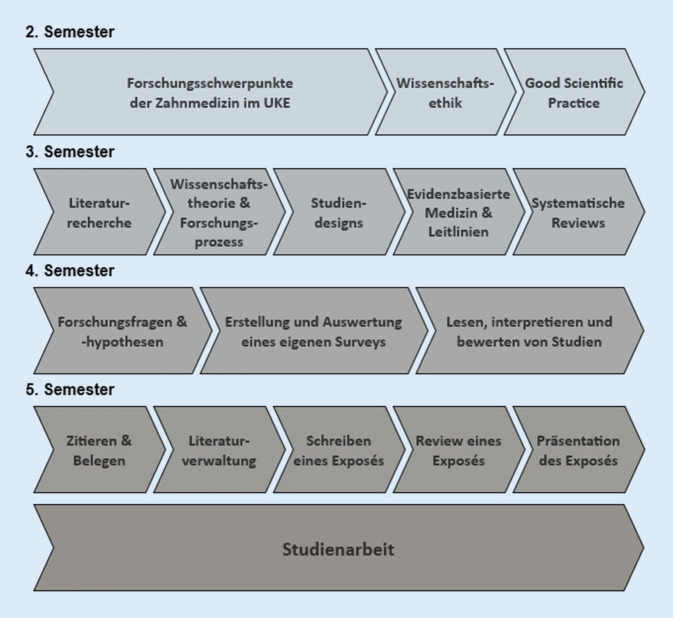

Der longitudinale Wissenschaftsstrang beginnt im 2. Semester und setzt sich kontinuierlich bis zur Umsetzung des Erlernten in der Studienarbeit im Modul S im 5. Semester fort (Abb. 2).

Bereits im Studienabschnitt „Normalfunktion“ erfolgt eine Einführung in die Forschungsschwerpunkte der zahnmedizinischen Kliniken. Zudem werden die Grundlagen von Wissenschaftsethik und Good Scientific Practice vorgestellt und durch die selbstständige Arbeit an Anwendungsbeispielen vertieft.

Im darauffolgenden Studienabschnitt „Vom Symptom zur Erkrankung“ werden die Grundlagen des wissenschaftlichen Arbeitens vermittelt. Im Modul D1 (3. Semester) werden die Prinzipien der Wissenschaftstheorie und des Forschungsprozesses vorgestellt. Zusätzlich erlernen die Studierenden die Instrumente der Literaturrecherche und die Verwendung medizinischer Forschungsdatenbanken für systematische Reviews. Nach einer Einführung in die Grundlagen epidemiologischer und klinischer Studien wird das Prinzip der evidenzbasierten Medizin anhand der parallel unterrichteten zahnmedizinischen Inhalte am Beispiel der S3-Leitlinie zu vollkeramischen Kronen und Brücken erläutert [4]. Ziel dieses Moduls ist es, dass sich die Studierenden unter Anleitung den aktuellen Forschungsstand zu einem relevanten zahnmedizinischen Patient:innenfall erarbeiten können.

Im Fokus des 4. Semesters stehen das Lesen und kritische Bewerten von zahnmedizinischen Studien, um Schlussfolgerungen in den zahnmedizinischen Alltag zu integrieren. Hierzu entwickeln die Studierenden eigene Forschungsfragen und -hypothesen im Rahmen eines selbst erhobenen Surveys in ihrer Studienkohorte. Für die Beantwortung dieser Hypothesen erlernen sie relevante statistische Grundbegriffe und wenden diese selbstständig an. Zusätzlich lesen und erarbeiten sich die Studierenden 2 Publikationen. Zuerst werden in der Vorlesung die Kriterien für eine methodisch anspruchsvolle Publikation anhand einer Beispielstudie vorgestellt und besprochen und in den anschließenden Seminaren an einer zweiten Publikation angewandt.

In der ersten Hälfte des 5. Semesters erfolgt die finale Vorbereitung auf die Studienarbeit. Die Studierenden erlernen das korrekte Zitieren, Belegen mit wissenschaftlichen Quellen und die Verwendung einer Literaturverwaltungssoftware. Im Anschluss werden ihnen die Grundlagen des Schreibprozesses und wissenschaftlicher Vorträge vermittelt. Die begleitenden Seminare sind als Schreibwerkstätten angelegt, in denen die Studierenden Zeit für die Erstellung des Exposés ihrer Studienarbeiten haben und dabei auf die Beratung der Seminarleitenden zurückgreifen können. Danach erfolgen ein paarweises Review und die Einarbeitung in das Exposé. Final präsentieren sie ihre Ergebnisse in der Seminargruppe und geben sich gegenseitig Feedback.

Durch die eigenständige Auseinandersetzung mit einem wissenschaftlichen Themengebiet und dem Verfassen einer Studienarbeit im Modul S schließt der longitudinale Wissenschaftsstrang mit der Prüfung einer akademischen Schlüsselqualifikation ab. Dabei sollen die Studierenden den Nachweis erbringen, dass sie in der Lage sind, innerhalb einer vorgegebenen Frist von 7 Wochen ein Thema aus dem jeweiligen Fach- bzw. Themengebiet selbstständig mit erworbenen wissenschaftlichen Methoden zu bearbeiten. Für die Themenwahl ihrer Studienarbeit können die Studierenden an die Lehrenden der medizinischen Fakultät herantreten. Die Themen können fachübergreifend gestellt werden.

Zur Beurteilung des Lernzuwachses der Studierenden wurde die studentische Selbsteinschätzung in Bezug auf einzelne Aspekte wissenschaftlichen Arbeitens jeweils am Ende des 2. bis 5. Semesters erhoben, die evaluierten Items sind in Tab. 2 dargestellt. Es zeigt sich, dass sich die Selbsteinschätzungen der Studierenden im 2. Semester zunächst im mittleren Bereich bewegten und sich bis zur Abgabe der Studienarbeit erhöhen (Tab. 2).

**Table 2:** 

Median (Interquartilsabstand)	Modul B3	Modul D1	Modul E1	Modul S
*Ich kann …*				
*… eine bearbeitbare wissenschaftliche Frage formulieren*	4 (3–5)	5 (4–5)	4 (3–5)	5 (4–6)
*… eine Literaturrecherche durchführen*	4 (3–5)	4 (4–5)	4 (3–5)	5 (5–6)
*… aus der gefundenen Literatur die für die Fragestellung relevanten Arbeiten selektieren*	4 (3–5)	4 (4–5)	4 (3–5)	5 (4–6)
*… die Güte von Literatur/Studien kritisch einschätzen*	4 (3–5)	4 (4–5)	4 (3–4,75)	4 (4–5)
*… die Inhalte einer wissenschaftlichen Arbeit präsentieren*	4 (3–5)	4 (4–5)	4 (3–5)	5 (4–6)
*… korrekt zitieren und referenzieren*	4 (3–5)	3 (1–4)	3 (1–4)	5 (4–6)

## Prüfungen

Durch die Modullabschlussprüfungen, die 2‑mal pro Semester stattfinden, die manuelle Fortschrittsprüfung sowie den OSCE (Objective Structured Clinical Examination) gibt es im Modellstudiengang im Vergleich zum RSG eine höhere Prüfungsfrequenz, die für eine kontinuierliche Kontrolle des Lernerfolgs sorgt.

In jedem präklinischen Modul des iMED DENT können die Studierenden maximal 100 Modulpunkte erreichen. Diese setzen sich in unterschiedlichen Relationen aus den Punkten für praktische Arbeiten in den Kursen und einer Modulabschlussklausur zusammen (siehe auch Beitrag von Bender et al. in diesem Themenheft).

Für klinische Tätigkeiten, wie z. B. das Assistieren bei Patient:innenbehandlungen bei höheren Semestern in Modul F1, die Befundaufnahme bei Patient:innen im Modul G1 und in allen klinischen Patient:innenkursen kommt mit „EPA“ (Entrustable Professional Activity) eine weitere Prüfungsform für das Erreichen der Modulpunkte zum Tragen [5, 6]. Mit EPA wird neben den theoretischen und praktischen Kompetenzen auch das ärztliche Verhalten der Studierenden gegenüber ihren Patient:innen beurteilt (siehe auch Beitrag von Mirzakhanian et al. in diesem Themenheft).

### Z1 Prüfung

**Figure 3:**
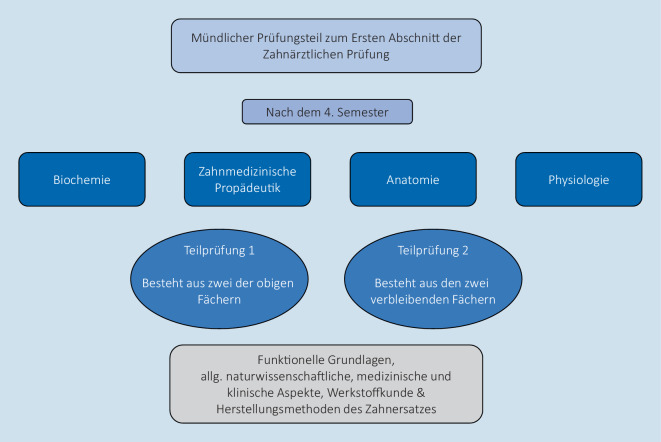

Die Z1 Prüfung besteht aus einem schriftlichen (Z1sÄ) und einem mündlichen (Z1mÄ) Teil und ist das Äquivalent zum ersten Abschnitt der zahnärztlichen Prüfung. Die Inhalte aus den Fächern Biologie, Physik und Chemie werden dazu in den Modulabschlussklausuren mitgeprüft (siehe auch Beitrag von Bender et al. in diesem Themenheft). Die Z1mÄ ist in Abb. 3 dargestellt.

### OSCE

OSCE ist eine standardisierte Prüfung klinisch relevanter Fähigkeiten der Studierenden, bei der an verschiedenen Stationen während eines definierten Zeitfensters Aufgaben aus dem Bereich der Medizin, Psychologie und Zahnmedizin absolviert werden müssen [7, 8]. Die Prüfung findet am Ende des Moduls D2 (Mitte des 6. Semesters) statt.

### Z2 Prüfung

**Figure 4:**
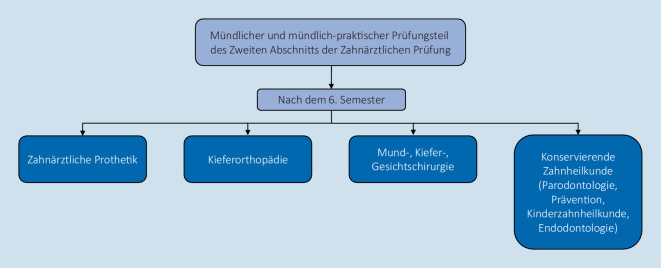

Diese Staatsprüfung zur „Behandlungsreife“ Z2 nach dem 6. Semester umfasst innerhalb von 2 Wochen praktische sowie theoretische Prüfungen in den zahnmedizinischen Fächern (Abb. 4). Die Bewertung erfolgt anhand der vom Institut für medizinische und pharmazeutische Prüfungsfragen (IMPP) konstruierten strukturierten Bögen. Auf diesen werden vorab von den Prüfenden Prüfungsfragen festgelegt und ein Erwartungshorizont definiert.

## Fazit

Die Reformziele des iMED DENT wurden im mittleren Studienabschnitt „Vom Symptom zur Therapie“ umfassend umgesetzt. Die bisherigen Evaluationen der Studierenden zeigen größtenteils eine hohe Zufriedenheit mit den Modulen dieses Studienabschnitts.

Eine kontinuierliche Anpassung und Optimierung der Interdisziplinarität der Fächer sind die Grundlage der stetigen Weiterentwicklung des Modellstudienganges. Dabei steht die Abstimmung der Studieninhalte auf die notwendigen Anforderungen und Kenntnisse der angehenden Zahnärzt:innen im Vordergrund, um sinnvolle Lehrinhalte zu intensivieren. Bei aller gewünschten Individualität des Ausbildungskonzeptes gilt es aber stets, die Vorgaben der Approbationsordnung und die Inhalte des Nationalen Kompetenzbasierten Lernzielkataloges als Grundlage zu berücksichtigen.

## Footnotes

1 Studienordnung Modellstudiengang Zahnmedizin (iMED Dent) vom 15.09.2021, 17.11.2021.https://www.uni-hamburg.de/campuscenter/studienorganisation/ordnungen-satzungen/pruefungs-studienordnungen/medizin/20211020-neuf-sto-med-zahnmedizin-100.pdf (Zugriff: 14.09.2023)

2 Studentische Lehrevaluation des Modell- und Regelstudiengangs der Zahnmedizin am UKE https://www.uke.de/studium-lehre/qualitaet-und-lehrservice/lehrevaluation/index.html (Zugriff: 14.09.2023)

## References

[1] Okuda Y, Bryson EO, DeMaria S (2009). The utility of simulation in medical education: what is the evidence?. Mt Sinai J Med.

[2] Ortwein H, Fröhmel A, Burger W (2006). Application of standardized patients in teaching, learning and assessment. Psychother Psychosom Med Psychol.

[3] Qureshi AA, Zehra T (2020). Simulated patient’s feedback to improve communication skills of clerkship students. BMC Med Educ.

[4] Meyer G, Ahsbahs S, Kern M (2014). Vollkeramische Kronen und Brücken. S3-Leitlinie.

[5] Arunachalam S, Pau A, Nadarajah VD, Babar MG, Samarasekera DD (2023). Entrustable professional activities in undergraduate dental education: A practical model for development and validation. Eur J Dent Educ.

[6] Ehlinger C, Fernandez N, Strub M (2023). Entrustable professional activities in dental education: a scoping review. Br Dent J.

[7] Eberhard L, Hassel A, Bäumer A (2011). Analysis of quality and feasibility of an objective structured clinical examination (OSCE) in preclinical dental education. Eur J Dent Educ.

[8] Schoonheim-Klein M, Walmsley AD, Habets L (2005). An implementation strategy for introducing an OSCE into a dental school. Eur J Dent Educ.

